# In memoriam - Peter Mariën (1962–2017)

**DOI:** 10.1186/s40673-017-0077-3

**Published:** 2017-12-19

**Authors:** Mario Manto, Alan Beaton, Roeland Crols, Philippe Paquier, Jo Verhoeven, Jeremy D. Schmahmann

**Affiliations:** 1 FNRS, ULB-Erasme, 808 Route de Lennik, 1070 Brussels, Belgium; 2 Service des Neurosciences, UMons, Mons, Belgium; 3 Human and Health Sciences, Swansea University, Swansea, UK; 4 Department of Neurology, ZNA Middelheim Hospital, Antwerp, Belgium; 5 Service de Neuropsychologie, ULB-Hôpital Erasme, Brussels, Belgium; 6 Research Unit of Clinical and Experimental Neurolinguistics, Vrije Universiteit Brussel (VUB), Brussels, Belgium; 7 Unit of Translational Neurosciences, Universiteit Antwerpen (UA), Antwerp, Belgium; 8 Division of Language and Communication Science, City, University of London, London, UK; 9 Ataxia Unit, Cognitive Behavioural Neurology Unit, Laboratory for Neuroanatomy and Cerebellar Neurobiology, Massachusetts General Hospital and Harvard Medical School, Boston, USA

**Keywords:** Peter Mariën, Professor, Neurolinguistics, Neurocognition, Cerebellum

**Fig. 1:**
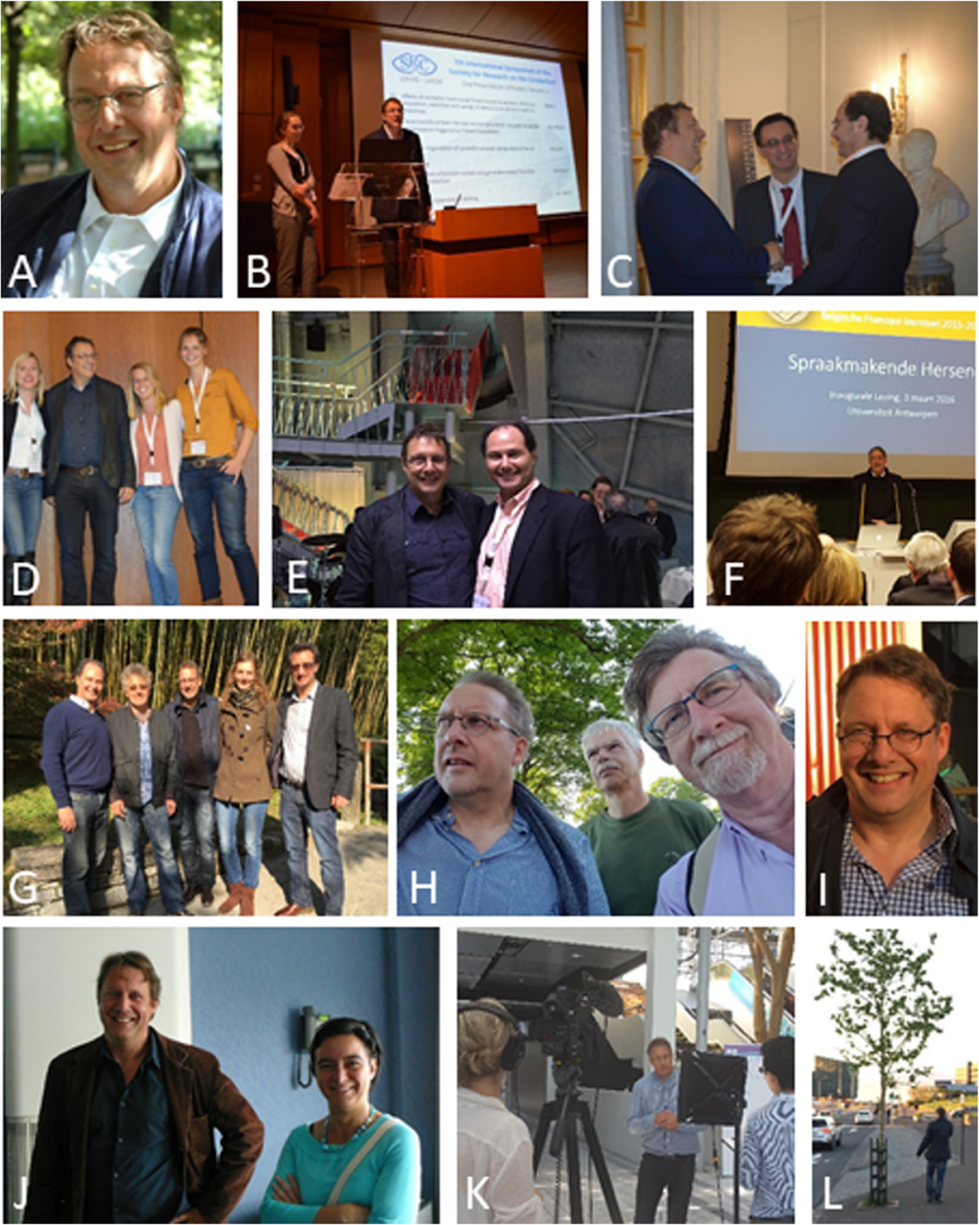
**a**. Photo of Peter Mariën (PM). **b**: PM during the International Symposium of SRC in Brussels (2015). **c**: PM on the left, Mario Manto (MM) in the centre, Jeremy Schmahmann (JS) on the right. **d**: Stefanie Keulen, PM, Elke De Witte, Kim van Dun (KvD) from left to right. **e**: PM and JS at the Atomium in Brussels. **f**: PM giving the Francqui lecture. **g**: JS, Frank van Overwalle, PM, KvD, MM from left to right. **h**: PM, Roeland Crols, Eric De Smet from left to right. **i**: PM in Iceland (2015). **j**: PM and Peggy Wackenier. **k**: PM during an interview. **l**: PM walking in Reykjavik. Permissions have been obtained from the copyright owners of the photos

The communities of cognitive behavioural neurology, neurolinguistics, and cognitive neuroscience of the cerebellum lost a true friend and stellar clinician-scientist in the premature passing of Professor Peter Mariën (Fig. 1) on November 1st, 2017. Peter was a vibrant and active member of the clinical and scientific communities in his university, his beloved Belgium, and in the international community. We lost him too soon, in the prime of his life and academic career, after a brief illness and a valiant and heroic fight.

Born in Geel (Belgium) on July 7th, 1962, he spent his childhood and attended school in the nearby small village of Meerhout. At the Universiteit Antwerpen (UA) (Belgium) he obtained his Bachelor of Arts in Linguistics in 1985, where he also earned his Master’s degree in 1987. He then studied neurolinguistics at the Vrije Universiteit Brussel (VUB) with Prof. Yvan Lebrun, one of the founders of neurolinguistics as a scientific discipline. He received his PhD in Medical Sciences from his Alma Mater (UA) in 2001, with a doctoral dissertation on crossed aphasia in dextrals. He further forged his career at the VUB, where in 2002 he was promoted to Professor of Neurolinguistics and Psycholinguistics, and in 2012 became Professor of Research Methodology in Linguistics and the Chair for Clinical and Experimental Neurolinguistics. He was a valued member of the Department of Neurology and Memory Clinic of ZNA Middelheim in Antwerpen. Since 2014, Peter had been Visiting Professor in Neurolinguistics at City University of London (UK). In recognition of his outstanding scientific career, Peter was awarded the Franqui Chair at the UA in 2016, at which time he delivered a series of highly appreciated lectures.

Peter Mariën’s field of expertise was clinical neurolinguistics, clinical neuropsychology and neurocognition. In the early 1990s he commenced his multi-disciplinary assessments of patients with neurobehavioural, neurocognitive and affective disorders following CNS damage. As his career progressed, he developed a special interest in and passion for studying and understanding cerebellar neurocognition [1–3]. Following the precedent of his PhD mentor, Luigi Vignolo, he performed meticulous and in-depth cognitive and neurobehavioural analyses of individual patients to advance our understanding of the functions of interconnected neural systems in health and disease. These careful expositions of neurobehavioural disorders included some of his favourites, such as crossed aphasia, apraxic agraphia, dyspraxia and foreign accent syndrome (FAS) following cerebellar lesions [4–7]. He was particularly interested in the mechanisms of FAS, a rare motor speech disorder described for the first time by the French neurologist Pierre Marie in 1907. He contributed significantly to a new taxonomy of the syndrome by making a fundamental distinction between neurogenic, psychogenic and mixed FAS. He worked out the concept of cerebellar-induced aphasia [8]. He championed the concept of the cerebellar cognitive affective syndrome (CCAS) in adults and children, including a developmental form, and its manifestation as part of what was previously called posterior fossa syndrome (now paediatric post-operative cerebellar mutism syndrome). Together with Mario Manto, Peter introduced, and insisted upon, the eponymous nomenclature for the CCAS [9]. He dissected disorders of social cognition and other neurobehavioural presentations of neurodegenerative diseases and stroke, and he was starting to explore approaches to therapy of these clinical phenomena, especially with the use of cerebellar transcranial direct current stimulation (tDCS). He was convinced that ataxiology is a major branch of the evolving tree of neurosciences, and he became an active Board Member of the international journal *Cerebellum* *and Ataxias* to promote the field.

His intellectual output was prolific and highly cited, with around 150 articles in peer-reviewed journals and 24 book chapters. In addition, he edited 4 books, including the most recent – *The Linguistic Cerebellum* [10]. He was a regular invited lecturer at international conferences, always delivering insightful, innovative, and thought-provoking material that helped advance the field. He organized and chaired international conferences that we have participated in, including meetings of the Society for Research on the Cerebellum and Ataxias, the working group on Aphasia and Cognitive Disorders of the World Federation of Neurology, and conferences dedicated to neurolinguistics and clinical aphasiology. He was instrumental in organizing special issues in international journals on cutting-edge topics [11]. He translated widely used neuropsychological and neurolinguistic tests into Dutch, and he was a beloved and highly sought-after teacher and a dedicated mentor at all levels. He taught neurological science to high school students, and led academic courses at universities throughout Belgium (Antwerp, Brussels, Ghent, Louvain), guiding a generation of students at VUB through the successful completion of their Master’s degrees and PhD dissertations, while maintaining a media presence through his exposition of instructive clinical cases and syndromes. Peter loved teaching and he was praised by his students for his excellent pedagogical skills. He inspired his students and colleagues with novel ideas, and he was the driving force for a large number of research projects. He was renowned for his aversion to bureaucratic paperwork and administrative shambles.

Those of us who knew him well, both at home and at meetings in Belgium and abroad, will miss Peter as much for his keen intellect and important contributions to academic discourse, as for his true friendship, his generosity of spirit, kind gentle nature, and caring attitude. He felt and showed genuine interest in the well-being of those around him. He had boundless enthusiasm, a great sense of humour, and deep respect for all those with whom he worked. His sense of fairness and decency was always in evidence, matched by his infectious smile and warm personality. Peter was devoted to his family and to his academic life, an avid reader who loved literature, the arts, biking, and travelling in Italy. He was always up for sharing a Duvel beer and Leonidas chocolates over meaningful conversation. He was the rare example of a gentleman and scholar, combining great personal warmth with genuine openness to fresh ideas. Peter was full of empathy, with a magnetic personality, tremendous energy and a general lust for life. He had a lively interest in all manner of things and people.

On behalf of the scientific community, and the cerebellum community in particular, we send our most heartfelt condolences to his wife Mieke Hens and their children, Marie and Louis. His legacy of scholarship, mentorship, and friendship lives on.
